# Current Treatment Options for REM Sleep Behaviour Disorder

**DOI:** 10.3390/jpm11111204

**Published:** 2021-11-14

**Authors:** Félix Javier Jiménez-Jiménez, Hortensia Alonso-Navarro, Elena García-Martín, José A. G. Agúndez

**Affiliations:** 1 Section of Neurology, Hospital Universitario del Sureste, Arganda del Rey, E-28500 Madrid, Spain; hortalon@yahoo.es; 2 University Institute of Molecular Pathology Biomarkers, Universidad de Extremadura, ARADyAL Instituto de Salud Carlos III, E-10071 Cáceres, Spain; elenag@unex.es (E.G.-M.); jagundez@unex.es (J.A.G.A.)

**Keywords:** REM sleep behaviour disorder, neurochemistry, neurotransmitters, dopaminergic dysfunction, noradrenalin, acetylcholine, synucleinopathies

## Abstract

The symptomatic treatment of REM sleep behaviour disorder (RBD) is very important to prevent sleep-related falls and/or injuries. Though clonazepam and melatonin are usually considered the first-line symptomatic therapy for RBD, their efficiency has not been proven by randomized clinical trials. The role of dopamine agonists in improving RBD symptoms is controversial, and rivastigmine, memantine, 5-hydroxytryptophan, and the herbal medicine yokukansan have shown some degree of efficacy in short- and medium-term randomized clinical trials involving a low number of patients. The development of potential preventive therapies against the phenoconversion of isolated RBD to synucleinopathies should be another important aim of RBD therapy. The design of long-term, multicentre, randomized, placebo-controlled clinical trials involving a large number of patients diagnosed with isolated RBD with polysomnographic confirmation, directed towards both symptomatic and preventive therapy for RBD, is warranted.

## 1. Introduction

The first reports of the disorder designated as “rapid eye movement (REM) sleep behaviour disorder” (RBD) were by Schenk et al. [1,2] in 1986. This new type of parasomnia, which was seen in patients with different neurological disorders (most of them were men), shared some similarities with a behavioural disorder found in cats with pontine tegmental lesions during REM sleep and was described as “abnormal behaviours during REM sleep such as stereotypical hand motions, reaching and searching gestures, punches, kicks, and verified dream movements”. Polysomnography (PSG), which is considered to be necessary for the definitive diagnosis of RBD [3], is characterized by a loss of chin atonia in variable degrees, increased REM ocular activity, increased REM limb-twitch activity, and increased density and duration of stage 3–4 slow-wave sleep.

According to their aetiology, RBD can be classified as “idiopathic” or “isolated” (iRBD; this diagnosis requires a lack of evidence of any diagnosed neurological disease) or secondary to narcolepsy, neurodegenerative diseases, drugs, or autoimmune disorders [4,5]. Structural lesions affecting the medulla, the pons, or the limbic system have also been reported as a cause of secondary RBD [6,7,8]. The long-term follow-up of patients initially classified as iRBD has shown the development of neurodegenerative diseases, mainly synucleinopathies such as Lewy body dementia (LBD), Parkinson’s disease (PD), or multisystem atrophy (MSA) [9]. Probable iRBD (that is, iRBD diagnosed on clinical grounds without PSG confirmation) is a high-prevalence disorder, with an estimated frequency of 5.65 (95% CI = 4.29–7.18%) according to a recent meta-analysis [10].

The aetiology of iRBD is largely unknown, although associations with an hexanucleotide repeat expansion in the C9orf72-SMCR8 complex subunit (*C9orf72*) gene [11], missense variations in the glucosylceramidase beta glucocerebrosidase (*glucocerebrosidase* or *GBA*) gene [12], and some rare variants in the bone marrow stromal cell antigen 1 (*BST1*) and lysosomal-associated membrane protein 3 (*LAMP3*) genes [13] have been described in some patients, and possible associations between smoking, farming, and previous head injuries to the risk for iRBD have been suggested [14].

The neurochemical features of RBD include dopaminergic deficiency (the most consistent finding) and changes in noradrenaline, acetylcholine, excitatory and inhibitory neurotransmitters, hormones such as melatonin, and proinflammatory factors [15]. Patterns resembling those of PD and/or LBD have been found in brain glucose metabolism and brain perfusion studies, and cortical grey matter atrophy, structural changes in deep grey matter nuclei, and alterations in the functional connectivity between several networks including the basal ganglia and cerebral cortex have been found by using structural and functional MRI [15].

Despite the assumption that the first-line drugs in the therapy or RBD are clonazepam and melatonin, an ideal treatment has not been established. The aim of this narrative review is to provide a description of studies reported to date related to the treatment of this clinical entity.

## 2. Search Strategy

The references used for this review were identified through a PubMed search that included the period from 1966 to 9 October 2021. The term “REM sleep behaviour disorder” was crossed with “treatment” (833 items), “therapy” (5 items), “pharmacology” (236 items), “neuropharmacology” (4 items), “clonazepam” (8 items), “melatonin” (115 items), “ramelteon” (8 items), “levodopa” (104 items) ”dopamine agonists” (55 items), “sodium oxybate” (15 items), “antidepressant drugs” (88 items), “antiepileptic drugs” (108 items), “cannabinoids” (6 items), and “herbals” (6 items). The whole search retrieved a total of 902 references that were manually examined. The final selection comprised 71 references strictly related to treatment options for RBD. The quality of reviewed works with level 1 of clinical evidence was assessed by applying a previously reported checklist of methodological items [16].

## 3. Clonazepam

The improvement of RBD symptoms in patients treated with clonazepam (a benzodiazepine that enhances the neurotransmitter gamma-aminobutyric acid (GABA) via the modulation of the GABA_A_ receptor and has antiepileptic properties) was suggested in the first description of RBD by Schenck et al. [1].

Table 1 summarizes the studies describing the effect of clonazepam in patients with RBD [1,2,17,18,19,20,21,22,23,24,25,26,27,28]. Surprisingly, despite clonazepam being considered a first-line therapy for RBD (and according to the experience of the authors of this review, it is a useful drug for treating this condition), its efficacy in the treatment of iRBD has apparently not been proven in randomized trials. As shown in Table 1, reports on the efficacy of clonazepam in iRBD should be classifiable as possessing levels II or III of evidence. In a randomized double-blind placebo-controlled clinical trial (level I of evidence, >50% of quality), Shin et al. [26] showed a similar degree of improvement in the RBD symptoms of PD patients with RBD between clonazepam at low doses (0.5 mg/day) and a placebo.

In a study involving 36 patients diagnosed with RBD treated with clonazepam as the first-line therapy, 58% developed moderate to severe side-effects, the most frequent being daytime sedation, confusion, and cognitive impairment [29]. It has been suggested that these side effects could increase the risk of falling and fall-related injuries in elderly patients [30]. In addition, it has been reported that clonazepam could induce or aggravate sleep apnoea syndrome in some patients [31].

Anderson et al. [29] reported the efficacy and tolerance of the cyclopyrrolone zopiclone (at 3.75–7.5 mg at night, alone or in combination, this drug increases GABAergic transmission by modulating benzodiazepine receptors) in 8 of 11 patients with side effects of clonazepam (level III of evidence) in an open-label study.

## 4. Melatonin and Its Analogues

Data from publications regarding the efficacy of the pineal hormone melatonin in RBD are summarized in Table 2 [21,27,32,33,34,35,36,37,38,39,40,41,42,43]. Melatonin secretion has been reported as delayed by 2 h in patients with RBD [44]. Together with clonazepam, melatonin is considered to be a first-line therapy for RBD.

Most reports suggesting improvements of RBD with melatonin have been single case reports, open-label trials, or retrospective analyses of cohorts (therefore classifiable as level II or level III of evidence) [21,27,32,33,34,35,36,37,38,39,40,43]. Only three studies were randomized clinical trials with level I of evidence and a quality rate >50%, one of them involving a short series of eight patients treated with 3 mg of melatonin at night who showed significant clinical and PSG improvement of RBD [37] and the other two involving 30 patients treated with 2–6 mg/day of prolonged release melatonin who both showed a lack of improvement of RBD [41,42].

The atypical antidepressant drug agomelatine acts as agonist of the melatonin receptors MT_1_ and MT_2_, and the antagonist of the serotonin (5-hydroxytryptamine or 5-HT) 5-HT_2C_ and 5-HT_2B_ receptors. Bonakis et al. [45] reported the total or partial improvement of RBD symptoms in three patients with iRBD (Table 3).

The efficacy of ramelteon, a melatonin receptor agonist with a high affinity for MT_1_ and MT_2_ receptors and selectivity over the MT_3_ receptor, in the treatment of RBD has been reported in two patients with RBD associated with MSA [46] and in an open-label trial of patients with PD (68.6% of them with concomitant RBD; level II of evidence) [47]. However, another open-label trial failed to find significant improvement in patients diagnosed with RBD [48]. The results of studies with agomelatine and ramelteon are summarized in Table 3.

## 5. Dopamine Acting Drugs

The results of studies addressing the possible efficacy of dopaminergic drugs in the treatment of RBD are summarized in Table 4. Improvements of RBD symptoms with levodopa in three patients with RBD and preclinical PD [49] and in a patient with LBD and concomitant RBD have been described [50].

While three open-label studies (level-II of evidence) [51,52,54] and a retrospective cohort involving 81 patients with iRBD (level-II of evidence) [55] showed a beneficial effect of pramipexole (a D_2_ and D3 non-ergoline dopamine receptor agonist with D_3_-preferring receptor-binding profile) in 60–80% of patients diagnosed with iRBD, another open-label study (level-II of evidence) showed a lack of improvement of RBD symptoms in 11 patients with PD and concomitant RBD treated with low doses of this drug as add-on therapy to levodopa [53]. In contrast, another open-label study (level-II of evidence) involving 11 patients with PD and RBD showed a beneficial effect of the dopamine agonist rotigotine (a non-ergoline and non-selective agonist of the dopamine D_1_, D_2_, D_3_, and (to a lesser extent) D_4_ and D_5_ receptors, with the highest affinity for the D_3_ receptor) at relatively higher doses [56].

On the other hand, a recent study involving 250 patients diagnosed with idiopathic PD who completed a RBD Screening Questionnaire (RBDSQ) [58] showed an association between RBDSQ scores and the doses of levodopa used (while no association was found with dopamine agonists), although it could not be excluded that this result may have been related to the PD duration and/or severity [59]. Previously, Ozekmekçi et al. [60] reported that, compared to PD patients without RBD, patients with RBD showed higher duration of the disease and higher current doses of levodopa, a finding that could suggest a relationship between the cumulative doses of levodopa and the development of RBD.

Finally, a recent longitudinal randomized cross-over study in with safinamide (a potent and selective monoamine oxidase B (MAOB) inhibitor that enhances dopaminergic neurotransmission and inhibits glutamate release and dopamine and serotonin reuptake) involving 30 patients with PD and RBD showed a significant clinical improvement of RBD symptoms in more than 70% [57].

## 6. Sodium Oxybate

Sodium oxybate, also named sodium 4-hydroxybutyrate and sodium 4-hydroxybutanoate, is the sodium salt of γ-hydroxybutyric acid, and it is used for the treatment of sudden muscle weakness and excessive daytime sleepiness in patients with narcolepsy. The results of reports on the treatment of RBD with sodium oxybate are summarized in Table 5. Some anecdotal reports described clinical improvements of RBD symptoms in patients with iRBD [61,62], RDB associated with PD [63], and narcolepsy type 1 [64]. A recent open-label trial involving 19 children and adolescents with RBD associated with narcolepsy type 1 (level II of evidence) also showed the clinical and PSG improvement of RBD symptoms [65].

## 7. Other Treatments

### 7.1. Drugs Used for the Therapy for Alzheimer’s Disease

Ringman and Simmons [66] reported the marked and prolonged improvement of RBD symptoms in three patients with AD and RBD after starting treatment with 10–15 mg/day of the cholinesterase inhibitor donepezil. In addition, two short-term double-blind crossover pilot studies (level I of evidence; >50% of quality score) showed improvements of RBD symptoms resistant to clonazepam and melatonin in patients with RBD associated with PD [67] and mild cognitive impairment [68], respectively, with 4.6 mg/day of the cholinesterase inhibitor rivastigmine (Table 6). Interestingly, a case of RBD induced by rivastigmine in a patient diagnosed with AD was reported [69].

A recent double-blinded placebo-controlled randomized multicentre trial (level I of evidence; quality score >50%; see Table 6) showed a beneficial effect of memantine [83]. This drug is a low-affinity, voltage-dependent, non-competitive antagonist of the glutamatergic N-methyl-D-aspartate (NMDA) receptors, a non-competitive antagonist of the 5-HT_3_ receptor, antagonist of several neuronal nicotinic acetylcholine receptors (nAChRs), and agonist of the D_2_ receptors.

### 7.2. Antidepressant and/or Serotoninergic Drugs 

The effects of antidepressants and/or serotoninergic drugs on RBD are summarized in Table 6. Some anecdotal reports described improvement of RBD with tricyclic antidepressants such as desipramine [1] and imipramine [70]; the selective serotonin reuptake inhibitors fluvoxamine [71] and paroxetine [71]; the serotonin antagonist and reuptake inhibitor trazodone [72]; the MT_1_ and MT_2_ receptor agonist and 5-HT_2C_ and 5-HT_2B_ receptor antagonist agomelatine [45]; and the serotonin reuptake inhibitor, 5-HT_1A_ and 5-HT_1B_ receptor agonist, 5-HT_1D_, 5-HT_3_, and 5-HT_7_ receptor antagonist, and likely ligand of the β_1_-adrenergic receptor vortioxetine [73].

Nelotanserin (a drug primarily developed for the treatment insomnia that acts as an inverse agonist on the serotonin receptor subtype 5-HT_2A_) at a dose of 80 mg/day was tested in a short-term double-blind placebo-controlled randomized multicentre trial (level I of evidence; >50% of quality score) in patients with RBD associated with DLB or PD-dementia (PDD); negative results were reported [74].

Finally, a recent short-term randomized double-blind placebo-controlled crossover trial showed a beneficial effect of 5-hydroxytryptophan (the precursor of serotonin) in the treatment of RBD symptoms in patients with PD and RBD (level I of evidence; >50% of quality score) [75].

### 7.3. Antiepileptic Drugs 

The effects of antidepressants and/or serotoninergic drugs on RBD are summarized in Table 6. Studies have reported marked improvements of RBD symptoms in one patient with iRBD treated with carbamazepine [76] and one patient with RBD associated with probable LBD treated with levetiracetam [77]. To our knowledge, there has been no report of possible improvements of RBD with lamotrigine, but the worsening of RBD symptoms in a patient diagnosed with iRBD related to lamotrigine withdrawal was described [84].

Potassium bromide (K Br), a salt that was widely used as an antiepileptic and sedative in the late 19th and early 20th centuries and is used as an antiepileptic medication for dogs, was shown to be effective in the treatment of RBD-like symptoms in 14 dogs [85]

### 7.4. Cannabinoids

The effects of antidepressants and/or serotoninergic drugs on RBD are summarized in Table 6. Cannabidiol is a phytocannabinoid that acts an antagonist of the cannabinoid CB_1_ and CB_2_ receptors, with a low affinity for them. Following the description of short-term improvements of RBD symptoms in four patients with PD and RBD [78], a randomized placebo-controlled clinical trial involving 33 patients with PD and RBD (level I of evidence; quality score >50%) showed improvement in sleep satisfaction but not significant differences in the control of RBD at two different doses of cannabidiol compared with placebo [79].

### 7.5. Herbals

The effects of antidepressants and/or serotoninergic drugs on RBD are summarized in Table 6. The herbal medicine yokukansan or Yi-Gan San has led to improvements of RBD symptoms in a short case series [80] and two retrospective analyses of clinical records [81,82] (in one of them via a comparison with clonazepam [82]).

### 7.6. Non-Pharmacological Therapies

Howell et al. [86] showed a decrease in RBD symptoms and sleep-related injuries in four patients diagnosed with RBD resistant to clonazepam and melatonin therapy by using customized bed alarms with a familiar voice to deliver a calming message at the onset of dream enactment behaviours (level III of evidence) based on complex auditory processing and the low arousal threshold of REM sleep.

Finally, McCarter et al. [87] speculated that high-intensity exercise could have a protective role in the development of Parkinsonism in patients initially diagnosed with iRBD based on the attenuation of the symptomatic progression of PD and the delayed onset of AD with high-intensity exercise in humans, as well as the demonstration of a reduction of accumulation of α-synuclein, tau protein, and amyloid beta in animals by exercise.

## 8. Discussion and Conclusions

The search for appropriate treatments for RBD is important for preventing sleep-related injuries of both patients and their partners. In addition, due to the high described rate of the phenoconversion of iRB to synucleinopathies, it is important to try potential preventive therapies following the early detection of patients at risk. However, the ideal therapy for RBD is not currently established. Traditionally, clonazepam and melatonin have been used as first-line treatments based on the descriptions of improvements of RBD symptoms of many patients treated with these drugs in single case reports, case series, retrospective medical reports, and open-label trials (Table 1 and Table 2). However, only one randomized clinical trial addressing the effects of clonazepam versus placebo in PD patients with RBD failed to find significative differences between clonazepam and placebo [26]; another randomized clinical trial involving a small number of patients that compared 3 mg of standard-release melatonin with placebo found a higher degree of improvement with melatonin, and two other shorter randomized clinical trials showed a similar efficacy of prolonged-release melatonin (2, 4, and 6 mg) compared to placebo in improving RBD symptoms [41,42].

The possible efficacy of analogues of melatonin (Table 3), levodopa, and dopamine agonists (Table 4) has not been established and is controversial, although a large retrospective cohort study (level II of evidence) suggested an improvement in 61.7% of iRBD non-responders to clonazepam with pramipexole therapy. Moreover, one must consider the possibility that levodopa at high doses could be related to the risk of developing RBD in PD patients [56]. Safinamide could be useful in the treatment of RBD associated with PD according to a recent longitudinal randomized clinical trial involving a small number of patients [57].

Results of randomized clinical trials, most of them involving a limited number of patients, have shown the short-term efficacy of the anticholinesterase drugs rivastigmine [67,68] and 5-hydroxytryptophan [75] and the herbal medicine yokukansan [81,82], as well as the lack of efficacy of nelotanserin [74] and cannabidiol [79]. Memantine has shown some degree of medium-term efficacy in the reduction of RBD symptoms in patients with LBD and dementia associated with PD [83].

Finally, the possible improvements reported with sodium oxybate [61,62,63,64,65], desipramine [1], imipramine [70], fluvoxamine [71], paroxetine [71], agomelatine [45], trazodone [72], vortioxetine [73], carbamazepine [76], levetiracetam [77], and cannabidiol [78] are limited to single case reports, short case series, or open-label studies.

The results of this review are, in general, in agreement with those of other previous reviews [88,89,90,91,92,93,94,95,96,97,98]. Ideally, the design of therapeutical trials for RBD should pursue two main types of objectives: the adequate symptomatic therapy for RBD symptoms and the possibility of developing neuroprotective (preventive) strategies. Symptomatic therapy should be developed to prevent sleep-related falls and injuries and to improve the quality of sleep of the patients, while the development of neuroprotective therapies, at least in patients with higher risks of developing synucleinopathies) could be used to delay the clinical onset and improve the clinical course of these diseases.

For both types of studies, the design should be prospective and multicentre, involved a large series of patients diagnosed with iRBD with polysomnographic confirmation, and possessing a large follow-up period. Studies on symptomatic therapies should be randomized, double-blind, and placebo-controlled, and they could include several active pharmacological branches including clonazepam, melatonin, and other drugs that have shown any possibility of improving RBD symptoms in non-controlled trials or in cohort studies. Follow-up evaluation should include sleep diary, well-validated scales for RBD symptoms, the use of actigraphy devices, and at least one basal PSG study at the start and end of the follow-up.

Given the facts that PD is a very common neurological disorder and RBD is frequently associated with it before the onset of motor symptoms, it seems reasonable studies looking for neuroprotective strategies should include a selection of patients with polysomonographically confirmed iRBD and neurochemical and/or neuroimaging markers that suggest a high-risk of phenoconversion to synucleinopathies [15,91,97,99]. The combination of functional neuroimaging studies using different tracers, transcranial sonography, brain perfusion and glucose metabolism studies, functional MRI, and the detection of α-synuclein in certain tissues should be useful for this purpose [15].

## Figures and Tables

**Table 1:** Studies describing effects of clonazepam in patients with RBD.

Authors, Year [Ref]	Study Setting/Design	Type of Study	Main Findings	Level of Evidence (Quality Score)
Schenck et al., 1986 [1]	The first description of 4 RBD patients	Case series	Considerable improvement with clonazepam in 2 patients	III (NA)
Schenck et al., 1989 [17]	Description of a series of 100 patients with sleep-related injuries (33 of the 36 patients with RBD were treated with 0.25–2 mg of clonazepam at bedtime)	Observational case series	Improvement of sleep-related injuries in the majority of patients treated with clonazepam	III (NA)
Schenck et al., 1993 [18]	Description of a series of 96 RBD patients, 67 of them treated with clonazepam	Observational case series	“Complete efficacy” of clonazepam in 79% of patients and “partial efficacy” in 13.4% of patients	II (NA)
Olson et al., 2000 [19]	Description of a series of 93 RBD patients, 57 of them treated with clonazepam (38 with available information)	Observational case series	Clonazepam treatment was “completely successful” in 55.2% of patients, “partially successful” in 31.6% of patients, and “unsuccessful” in 13.1% of patients	II (NA)
Ferri et al., 2013 [20]	Comparison of CGI-S, RBDSS, and atonia index using video-PSG recording in 15 RBD patients under clonazepam treatment and 42 untreated patients	Observational case-control study	Patients treated with clonazepam showed a lower rate of stage shifts, higher sleep efficiency, a lower percentage of wakefulness after sleep onset and of sleep stage 1, an increased percentage of sleep stage 2, and improvement in CGI-S but similar RBDSS and atonia index	III (NA)
McCarter et al., 2013 [21]	Description of a series of 45 RBD patients (60% reported RBD-associated injury before treatment); 18 of them were treated with clonazepam	Retrospective cohort study	RBD VAS, RBD injuries, and falls from the bed improved significantly with clonazepam (less than with melatonin); 22% of patients discontinued clonazepam because of side-effects	II (NA)
Ferri et al., 2013 [22]	Comparison of RBDSS, atonia index, and NREM sleep instability using video-PSG recording in 15 iRBD patients, 13 narcolepsy/RBD patients, and 18 normal controls. Re-evaluation of iRBD patients was conducted 2.75 ± 1.62 years after treatment with 0.5–1 mg of clonazepam	Longitudinal follow-up study	RBD patients showed increased slow transient and decreases fast transient EEG events, reduced atonia index during REM sleep, and increased atonia index during NREM sleep Long-term therapy with clonazepam decreased WASO, increased slow-wave sleep and sleep stage 2, decreased sleep stages 1 and 2 instability, and decreased the duration of EEG transients, without modifying chin tone	II (NA)
Fernández-Arcos et al., 2016 [23]	Description of a series of 203 RBD patients, 167 of them treated initially with clonazepam	Observational case series	Clonazepam treatment was “completely successful” in 55.1%.	II (NA)
Li et al., 2016 [24]	Clinical (including modified RBDQ-3M) before and 28.8 ± 13.3 months after the initiation of treatment with clonazepam in 39 iRBD patients	Longitudinal follow-up study	“Treatment response” (complete elimination of sleep-related injuries and potentially injurious behaviours) in 66.7% of patients. Significant reduction of disturbing dreams with violent and frightening content. An increase in REM-related EMG activities was observed at follow-up. Less optimal treatment outcomes were associated with comorbid obstructive sleep apnoea and earlier onset of RBD	III (NA)
Ferri et al., 2017 [25]	29 drug-naïve iRBD patients, 14 iRBD patients treated with clonazepam, and 21 controls.Quantitative measurement of power spectra values of each REM sleep EEG spectral band using REM sleep EEG	Observational case-control study	The normalized power values a less pronounced REM-related decrease of power in all bands with frequency <15 Hz and an increase in the beta band (that was negatively correlated with muscle atonia) in drug-naïve iRBD patients, compared with controls. iRBD patients treated with clonazepam showed a partial return of all bands <15 Hz toward the control values	II (NA)
Shin et al., 2019 [26]	40 patients with PD and clinically diagnosed RBD treated with 0.5 mg/day of clonazepam at bedtime (*n* = 20) or placebo (*n* = 20) Assessment of CGI-I, KESS, PDSS, KV-MoCA, and UPDRS.	Four-week, randomized, double-blind, placebo-controlled trial	CGI-I score showed an improvement in RBD symptoms in patients treated with clonazepam that did not significantly differ from that of patients under placebo Secondary outcomes (KESS, PDSS, KV-MoCA, and UPDRS) did not significantly differ between the clonazepam and placebo groups	I (>50%)
Sunwoo et al., 2020 [27]	Assessment of “treatment response” (“presence or absence of any improvement in dream-enacting behaviours or unpleasant dreams after treatment”) in 123 iRBD patients treated with clonazepam (*n* = 40), melatonin (*n* = 56), and clonazepam-associated with melatonin (*n* = 27)	Retrospective review of medical records	Ninety-six patients (78.0%) reported improvement in their RBD symptoms during a mean follow-up period of 17.7 months RBDQ-KR, PSQI, SCOPA-AUT, and KVSS scores, as well as frequency or excessive daytime sleepiness, did not differ significantly between responders and non-responders Depression was significantly more frequent in non-responders	II (NA)
Lee et al., 2021 [28]	Assessment of “treatment response” (complete cessation of disruptive behaviours that may result in sleep-related trauma) in 171 PSG-confirmed RBD patients treated with clonazepam alone (*n* = 147) or in combination with other drugs (*n* = 24; 18 carbamazepine, 3 zolpidem, and 1 melatonin)	Retrospective review of medical records of patients with follow-up longer than 18 months (57.9 + 35.6 months)	One-hundred and fifty-five patients (90.6%) showed a positive response to the treatment positively, with an average dose of clonazepam of 1.01 + 0.49 mg at the last visit 24 patients experienced side effects of clonazepam (13 drowsiness, 6 dry mouth, 3 constipation, 1 erectile dysfunction, and 1 cloudiness) Earlier age at onset and at diagnosis and the presence of comorbid periodic limb movement during sleep were associated with poor treatment response	II (NA)

CGI-I, clinical global impression-improvement; CGI-S, clinical global impression-severity; EEG, electroencephalography EMG, electromyography; iRBD, idiopathic or isolated REM sleep behaviour disorder; KESS, Korean Epworth Sleepiness Scale; KV-MoCA, Korean Version of the Montreal Cognitive Assessment; KVSS, Korean version of the sniffin’ stick; NA, not applicable; NREM, non-rapid eye movements; PD, Parkinson’s disease; PDSS, Parkinson’s Disease Sleep Scale; PSG, polysomnography; PSQI, Pittsburgh Sleep Quality Index; RBD REM, sleep behaviour disorder; RBDQ-KR, RBD Questionnaire-Korean version; RBDQ-3M, modified RBD Questionnaire; RBDSS, RBD severity score, REM, rapid eye movements; SCOPA-AUT, Scales for Outcomes in Parkinson’s Disease Autonomic; SWS, slow-wave sleep; UPDRS, Unified Parkinson’s Disease Rating Scale; VAS, visual analogue scale; WASO, wake after sleep onset.

**Table 2:** Studies describing effects of melatonin in patients with RBD.

Authors, Year [Ref]	Study Setting/Design	Type of Study	Main Findings	Level of Evidence
Kunz and Bess, 1997 [32]	One patient with RBD treated with melatonin	Single case report	The first description of improvement of RBD with 3 mg/day of melatonin (significant reduction of motor activity during sleep measured by actigraphy and improvement in REM-sleep atonia by PSG)	III (NA)
Kunz and Bess, 1999 [33]	Six consecutive RBD patients were treated over 6 weeks with 3 mg of melatonin 30 min before bedtime. Clinical and PSG evaluation	Open-label trial	Dramatic clinical improvement in five of six patients within a week up to the end of treatment. Normalization of the percentage of REM sleep, a significant reduction of REM sleep without muscle atonia, a significant reduction of stage shifts in REM, and a significant reduction in epochs considered to be movement time in REM by PSG.	III (NA)
Takeuchi et al., 2001 [34]	15 PSG confirmed RBD patients treated with 3–9 mg/day of melatonin. Clinical and PSG evaluation, measurement of blood melatonin levels	Open-label trial	Marked, moderate, and mild improvement in 3, 9, and 1 patients, respectively. Two patients did not report improvement. Significant reduction of tonic REM activity in PSG after treatment with melatonin, with other PSG parameters remaining unchanged.	II (NA)
Boeve et al., 2003 [35]	14 patients with secondary RBD treated with 3–12 mg/day of melatonin because of lack of response to (*n* = 6) or severe side-effects with clonazepam (*n* = 2), cognitive impairment (*n* = 6), or presence of severe obstructive sleep apnoea (*n* = 1) and narcolepsy (*n* = 1)	Open-label trial. In 7 patients, melatonin was used as add-on therapy to 0.5–1 mg/day of clonazepam	Symptomatic control in 6 patients, significant improvement in 4, initial improvement with further worsening in 2, lack of improvement in 1, and increased severity of RBD in 1. Effective melatonin doses: 3 mg (*n* = 2), 6 mg (*n* = 7), 9 mg (*n* = 1), and 12 mg (*n* = 2) Side-effects in 5 patients resolved with decreasing doses (2 with morning headaches or sleepiness and 1 with delusions/hallucinations. Eight patients continued experiencing therapeutic improvement after 12 months of therapy	II (NA)
Anderson et al., 2008 [36]	Single case report	Single case report	Description of improvement of RBD in a patient diagnosed Alzheimer’s disease with 10 mg/day of melatonin (nearly complete resolution with no further episodes of self-injury or injury to his wife)	III (NA)
Kunz and Mahlberg, 2010 [37]	Eight male RBD patients treated with 3 mg of melatonin vs. placebo. Clinical (CGI-I) and PSG evaluation	Two-part, randomized, double-blind, placebo-controlled cross-over study	Significant improvement in CGI-I and reduction of the number of 30-s REM sleep epochs without muscle atonia during melatonin treatment. The number of REM sleep epochs without muscle atonia remained lower in patients who took placebo during Part II after having received melatonin in Part I, while patients who took placebo during Part I showed improvements in REM sleep muscle atonia only during Part II (melatonin treatment).	I (>50%)
McCarter et al., 2013 [21]	Description of a series of 45 RBD patients, (60% reported RBD-associated injury before treatment); 25 of them were treated with melatonin	Retrospective cohort study	RBD VAS and RBD injuries and falls from the bed significantly improved with melatonin (more than with clonazepam); 28% of patients discontinued clonazepam because of side-effects	II (NA)
Lyashenko et al. [38]	30 PD patients with PSG confirmed RBD. Treatment with 3–6 mg of melatonin ad bedtime for 4 weeks.	Open-label trial	84% of patients reported improvement of RBD symptoms	II (NA)
Schaefer et al., 2017 [39]	Four patients with RBD and concomitant obstructive sleep apnoea syndrome. Treatment with 2 mg of prolonged-release melatonin. Clinical and PSG evaluation.	Open-label study	Important clinical improvement. Non-significant changes in the percentage of REM sleep without atonia (attributed to the lack of therapy for obstructive sleep apnoea syndrome).	II (NA)
Kunz et al., 2017 [40]	A 72-year-old man diagnosed with Parkinson’s disease with reduced striatal DAT developed a typical RBD confirmed by PSG. Treatment with 2 mg of prolonged-release melatonin.	Single case report	Video-assisted PSG confirmed the diagnosis of RBD in 2012. Gradual improvement of clinical signs of RBD in 6 months and normalization of REM sleep with atonia two years later. Normalization of further DAT scans.	III (NA)
Jun et al., 2019 [41]	30 patients with PSG-confirmed iRBD. Treatment with prolonged-release 2 mg/day of melatonin, 6 mg/day of melatonin, or placebo 30 min before bedtime.Assessment with CGI-I and RBDQ-KR. The secondary outcomes included PSQI, ESS, SFRHS2 scores, as well as a sleep diary	A four-week, randomized, double-blind, placebo-controlled pilot study	Non-significant differences in the proportions of patients with a much or very much improved CGI-I score among the study groups. Non-significant differences in RBDQ-KR, PSQI, ESS, and SFRHS2, as well as in the sleep diary, among the groups.	I (>50%)
Gilat et al., 2020 [42]	30 PD patients with RBD. Treatment with 4 mg of prolonged-release melatonin or matched placebo at bedtime. Weekly diary or RBD incidents and adverse events.	Randomized, double-blind, placebo-controlled, parallel-group trial with an 8-week intervention and 4-week observation pre- and post-intervention	No differences between groups in events/week Similar adverse events between groups (mild headaches, fatigue, and morning sleepiness in 4 patients on melatonin and 5 on placebo).	II (>50%)
Sunwoo et al., 2020 [27]	Assessment of “treatment” response (“presence or absence of any improvement in dream-enacting behaviours or unpleasant dreams after treatment”) in 123 iRBD patients treated with clonazepam (*n* = 40), melatonin (*n* = 56), and clonazepam associated with melatonin (*n* = 27).	Retrospective review of medical records	Ninety-six (78.0%) patients reported improvement in their RBD symptoms during a mean follow-up period of 17.7 months RBDQ-KR, PSQI, SCOPA-AUT, and KVSS scores, as well as the frequency or excessive daytime sleepiness, did not significantly differ between responders and non-responders. Depression was significantly more frequent in non-responders.	II (NA)
Kunz et al., 2021 [43]	209 consecutive iRBD patients (171 patients had taken 2 mg of melatonin at 10–11 pm for ≥6 months, 13 had taken such for 1–3 months, and 25 used mixed treatments). Clinical evaluations with CGI and a newly developed RBD symptom severity scale (Ikelos-RS)	Single-centre, observational cohort study	RBD symptom severity gradually improved over the first 4 weeks of treatment and remained stably improved (mean follow-up 4.2 ± 3.1 years) The initial response was slowed to up to 3 months in patients taking beta-blockers or antidepressants and in patients with inadequately timed melatonin intake. When melatonin was discontinued after 6 months, RBD symptoms remained stably improved. When administered for only 1–3 months, RBD symptoms gradually returned.	II (NA)

CGI, clinical global impression; CGI-I, clinical global impression-improvement; DAT, dopamine transporter; ESS, Epworth Sleepiness Scale; iRBD, idiopathic or isolated REM sleep behaviour disorder; KVSS, Korean version of sniffin’ stick; NA, not applicable; PSG, polysomnography; RBD REM, sleep behaviour disorder; RBDQ-KR, RBD Questionnaire-Korean version; PSQI, Pittsburgh Sleep Quality Index; REM, rapid eye movements; SCOPA-AUT, Scales for Outcomes in Parkinson’s Disease Autonomic; SFRHS2, Short Form Health Survey version 2; VAS, visual analogue scale.

**Table 3:** Studies describing effects of melatonin analogues in patients with RBD.

Authors, Year [Ref]	Study Setting/Design	Type of Study	Main Findings	Level of Evidence (Quality Score)
Bonakis et al., 2012 [45]	3 patients with iRBD treated with agomelatine (MT1 and MT2 melatonin receptor agonist and a 5-HT_2_ antagonist) 25–50 mg 1 h before bedtime	Case report series	Significant improvement in 1 patient and partial improvement in 2 patients with 25 mg (these patients considerably improved with increasing dose to 50 mg) A trend for improvement in sleep efficiency with decreased WASO, a slight increase in the percentage of REM epochs with muscle atonia, and a decrease in 2 patients of the percentage of REM epochs with high tonic density No adverse effects were reported.	III (NA)
Nomura et al., 2013 [46]	The first description of 2 patients with PD and MSA treated with ramelteon (MT1 and MT2 melatonin receptor agonist)	Case report series	Improvement of clinical RBD symptoms Significant decrease in the percentage of REM sleep without atonia by PSG	III (NA)
Kashihara et al., 2016 [47]	35 patients diagnosed with idiopathic PD accompanied by sleep disturbances (24 of them with probable RBD) treated with 8 mg of ramelteon before sleep	A 12-week multicentre open-label trial	Significant improvement in RBDQ-JP, UPDRS Part III, and PDSS-2 scores	II (NA)
Esaki et al., 2016 [48]	12 patients with RBD treated with 8 mg of ramelteon 30 min before bedtime	4-week open-label trial	A non-significant trend toward clinical improvement in a visual analogue scale in some cases. Lack of significant effect on REM sleep without atonia or in an RBD severity scale measured by video-supported PSG.	II (NA)

5-HT, 5-hydroxytryptamine (serotonin); iRBD, idiopathic or isolated REM sleep behaviour disorder; MSA, multisystem atrophy; MT, melatonin; PD, Parkinson’s disease; PDSS-2, Parkinson’s disease Sleep Scale-2; PSG, polysomnography; RBD, REM sleep behaviour disorder; RBDQ-KR, RBD Questionnaire-Korean version; RBDQ-JP, RBD Questionnaire Japanese version; RBDSS, RBD severity score, REM, rapid eye movements; UPDRS, Unified Parkinson’s Disease Rating Scale; WASO, wake after sleep onset.

**Table 4:** Studies describing effects of levodopa and dopamine agonists in patients with RBD.

Authors, Year [Ref]	Study Setting/Design	Type of Study	Main Findings	Level of Evidence (Quality Score)
Tan et al., 1996 [49]	3 patients with iRBD preceding PD treated with levodopa (doses not stated)	Case report series	Dramatic clinical improvement of RBD symptoms in 2 patients and moderate improvement in 1 patient.	III (NA)
Yamauchi et al., 2003 [50]	1 patient with RBD as the initial symptom of DLB	Single case report	Clinical improvement of RBD symptoms, reduction of night time activity measured by actigraphy; a lack of muscle atonia and intermittent appearance of augmented muscle tone during REM sleep by PSG after treatment with levodopa, starting at 100 mg daily and increased to 500 mg daily	III (NA)
Fantini et al., 2003 [51]	8 patients diagnosed with iRBD were treated with 0.5–1 mg of pramipexole 1 h before bedtime. Clinical, video recording, and PSG assessment	Open-label study	Sustained reduction in the frequency or intensity of sleep motor behaviours (confirmed by video recording) in 5 patients. Lack of changes for the percentage of phasic EMG activity during REM sleep. Surprisingly, a decrease in the percentage of time spent with REM sleep muscle atonia	II (NA)
Schmidt et al., 2006 [52]	10 patients with PSG confirmed iRBD (6 of them with concomitant RLS or PLMS) treated with pramipexole (a single dose before bedtime or a divided dose regimen with the first dose given in the early evening and the second dose at bedtime). Clinical assessment with a mean follow-up of 13.1 months.	Open-label study	The average total evening dose of pramipexole at the end of the study was 0.89 + 0.31 mg. A divided dose regimen of pramipexole was used in 56% of patients remaining on pramipexole. 89% of patients experienced either a moderate or complete reduction in the frequency of RBD symptoms. 67% reported at least a moderate reduction in the severity of RBD symptoms.	II (NA)
Kumru et al., 2008 [53]	11 PD patients with RBD under stable dose of levodopa. Evaluation of the effect of pramipexole at an initial dose of 0.18 mg 3 times daily on RBD symptoms with bed partner interviews and blind assessment of video-PSG measures	Prospective open-label study	Lack of improvement of RBD-related symptoms and objective video-PSG abnormalities	II (NA)
Sasai et al., 2012 [54]	15 patients with iRBD with a PLMS index > 15 events/h shown. Treatment with 0.125–0.375 mg of pramipexole. PSG measures before and after 1 month of treatment.	Open-label study	Symptomatic improvement in 14 patients (80%) Reduction of REM density and PLMS index during non-REM sleep despite the unchanged amount of REM sleep without atonia. Positive correlation between the rate of change in RBD symptoms and the rate of REM density reduction.	II (NA)
Sasai et al., 2013 [55]	98 patients with iRBD treated with pramipexol (*n* = 81; in 31 non-responders, clonazepam was added) and/or clonazepam (*n* = 17; in 2 non-responders, pramipexol was added) during >3 months. Examination of PSG factors associated with pramipexole effectiveness	Retrospective cohort study	Efficacy of pramipexole in 61.7% (50/81) of patients Association of the ratio of REM sleep without atonia with total REM sleep with pramipexole effectiveness (with a cut-off rate of 16.8%). Responders to combined treatment with pramipexole and clonazepam showed significantly more REM sleep without atonia, total REM sleep, and frequency of vocalization or dream enactment behaviour than responders to monotherapy with pramipexole of with clonazepam	II (NA)
Wang et al., 2016 [56]	11 PD patients with untreated RBD. Administration of rotigotine at increasing doses (12.36 ± 4.27 mg at the end of the study; 24.7 ± 2.41 weeks). Evaluation of RBD symptoms through patient and bed partner interviews, RBDQ-HK, and blinded assessments of video-PSG measures	Prospective open-label study	Improvement of Parkinsonism and subjective sleep quality. Decrease in the RBDQ-HK total score, especially in the frequency and severity of abnormal RBD-related motor behaviours. Increase of total sleep time and stage 1 and decrease in PLMS index in the video-PSG without significant differences in RBD-related sleep measures	II (NA)
Plastino et al., 2021 [57]	30 patients with PD and RBD under stable antiparkinsonian therapy. Addition or no addition of safinamide 50 mg/day during 3 months, 15 days of washout, and switch of safinamide during other 3 months. Clinical (including PDSS-2 and RBDQ-HK scores) and PSG assessment	Longitudinal randomized cross-over study	Symptomatic improvement in 22 (73.3%) of patients (16 of them free from RBD symptoms at the end of treatment) Significant reduction in PDSS-2 and RBDQ-HK scale scores with safinamide. Significant increase of total sleep time and improvement in tonic submental electromyography activity, phasic submental electromyography activity, and REM density in the PSG study	I (>50%)

DLB, dementia with Lewy bodies; EMG, electromyography; iRBD, idiopathic or isolated REM sleep behaviour disorder; PD, Parkinson’s disease; PDSS-2, Parkinson’s disease Sleep Scale-2; PSG, polysomnography; PLMS, periodic leg movements during sleep; RBD, REM sleep behaviour disorder; RBDQ-HK, RBD Questionnaire-Hong-Kong version; REM, rapid eye movements; RLS, restless legs syndrome.

**Table 5:** Studies describing effects of sodium oxybate in patients with RBD.

Authors, Year [Ref]	Study Setting/Design	Type of Study	Main Findings	Level of Evidence (Quality Score)
Shneerson, 2009 [61]	One patient with iRBD treated with sodium oxybate resistant to multiple therapies (clonazepam, temazepam, zopiclone, melatonin, gabapentin, and clonidine)	Single case report	Long-term clinical improvement of RBD symptoms (at least 1 year) documented by video with 4.5 mg of sodium oxybate at night.	III (NA)
Liebenthal et al., 2016 [63]	One patient with RBD associated with PD treated with sodium oxybate who was resistant to multiple therapies (clonazepam, melatonin, prazosin, ramelteon, cyproheptadine, and eszopiclone)	Single case report	Long-term clinical improvement of RBD symptoms with 2.5 mg of sodium oxybate twice at night.	III (NA)
Mayer, 2016 [64]	One patient with RBD associated with narcolepsy type 1 treated with sodium oxybate	Single case report	Clinical improvement of RBD symptoms with 4.5 mg of sodium oxybate twice at night, with a reduction in muscle activity during REM sleep from 12.8% to 0%	III (NA)
Moghadam et al., 2017 [62]	Two patients with iRBD treated with sodium oxybate resistant to clonazepam alone or associated with carbamazepine, lamotrigine, melatonin, or pramipexole	Case report series	Treatment with 4.5 mg of sodium oxybate at night in one patient and 2.5 mg of sodium oxybate twice at night plus 2 mg of clonazepam in another resulted in long-term improvement of RBD symptoms assessed by bed-partner reports, VAS for frequency and severity, CGI-I scale and efficacy index, video-PSG, and at-home actigraphy.	III (NA)
Antelmi et al., 2021 [65]	19 children and adolescents with RBD associated with narcolepsy type 1. Treatment with 6.4 ± 1.2 g of sodium oxybate at night. Clinical and PSG assessment.	3 month open-label study	Marked clinical improvement in RBD symptoms. Decrease in ESS score Significant improvement of REM sleep atonia index and complex movements during REM sleep, decrease in the percentage of NREM sleep stage 1 and REM sleep, and increase in NREM sleep stage 3.	II (NA)

CGI-I, clinical global impression-improvement; ESS, Epworth Sleepiness Scale; iRBD, idiopathic or isolated REM sleep behaviour disorder; NREM, non-rapid eye movements; PSG, polysomnography; RBD, REM sleep behaviour disorder; REM, rapid eye movements; VAS, visual analogue scale.

**Table 6:** Description of the effect of other drugs in the treatment of RBD.

Drug	Authors Year, [Ref]	Study Setting	Type of Study	Main Findings	Level of Evidence (Quality Score)
**Drugs used in Alzheimer’s disease**					
Donepezil	Ringman and Simmons, 2001 [66]	3 patients with RBD treated with 10–15 mg/day of donepezil (one of them diagnosed with Alzheimer’s disease)	Case series	Marked improvement of RBD symptoms with donepezil (in two cases maintained for at least one year)	III (NA)
Rivastigmine	Di Giacopo et al., 2021 [67]	12 patients with PD (non-demented) and RBD confirmed by PSG resistant to clonazepam and melatonin, treated with 4.6 mg/day of rivastigmine or placebo	3-week, double-blind placebo-controlled, crossover pilot trial	Significant decrease of RBD episodes with rivastigmine compared to basal evaluation and placebo	I (>50%)
	Brunetti et al., 2014 [68]	25 patients with mild cognitive impairment and RBD confirmed by PSG resistant to clonazepam and melatonin treated with 4.6 mg/day of rivastigmine or placebo	30 days, placebo-controlled, cross-over pilot trial	Marked decrease of RBD episodes with rivastigmine compared to basal evaluation and placebo. 72% of patients treated with rivastigmine vs 20% with placebo showed at least 50% of reduction of RBD episodes.	I (>50%)
Memantine	Larsson et al., 2010 [69]	42 patients with DLB or PDD (probable RBD was assessed by a single question in the Stavanger Sleep Questionnaire). Treatment with 20 mg/day of memantine (*n* = 25) or placebo (*n* = 22)	24-week, double-blinded, placebo-controlled randomizedmulticentre trial	Frequency of moderate/severe probable RBD decrease from 44% to 13% in patients treated with memantine; this increased from 25% to 32% in the placebo group.	I (>50%)
**Antidepressant and/or serotonergic drugs**					
Desipramine	Schenck et al., 1986 [1]	The first description of 4 RBD patients	Case series	Considerable improvement of RBD symptoms with desipramine in 1 patient	III (NA)
Imipramine	Patterson et al., 1989 [70]	One patient with RBD treated with 75 mg of imipramine at bedtime (no improvement with 0.5 mg of clonazepam)	Single case report	Considerable improvement of RBD symptoms with imipramine maintained for at least 6 months	III (NA)
Fluvoxamine/paroxetine	Takahashi et al., 2008 [71]	One patient with RBD treated with 50 mg of fluvoxamine or 10 mg of paroxetine at bedtime	Single case report	Marked improvement of RBD symptoms with both fluvoxamine (withdraw because of bad tolerance) and paroxetine	III (NA)
Agomelatine	Bonakis et al., 2012 [45]	3 patients with iRBD treated with 25–50 mg of agomelatine (MT1 and MT2 melatonin receptor agonist and a 5-HT_2_ antagonist) 1 h before bedtime	Case series	Significant improvement in 1 patient and partial improvement in 2 patients with 25 mg of agomelatine (these patients considerably improved with increasing dose to 50 mg) A trend for improvement in sleep efficiency with decreased WASO, a slight increase in the percentage of REM epochs with muscle atonia, and a decrease in 2 patients of the percentage of REM epochs with high tonic density No adverse effects were reported.	III (NA)
Trazodone	Chica-Urzola, 2015 [72]	One patient with iRBD resistant to clonazepam treated with 50 mg/day of trazodone	Single case report	Marked improvement of RBD symptoms maintained for at least 3 months.	III (NA)
Vortioxetine	Du et al., 2020 [73]	One patient with RBD resistant to paroxetine and melatonin treated with 10 mg of vortioxetine.	Single case report	Marked improvement of RBD symptoms maintained for at least 3 months.	III (NA)
Nelotanserin	Stefani et al., 2021 [74]	26 patients with DLB and 8 with PDD with PSG-confirmed RBD. Treatment with 80 mg of nelotanserin or placebo (1:1 ratio). Assessment with video-PSG	4-week double-blind placebo-controlled randomizedmulticentre trial treatment period	Non-significant differences between nelotanserin and placebo in RBD behaviours.	I (>50%)
5-hydroxy-tryptophan (5-HTP)	Meloni et al., 2021 [75]	18 patients with PD and PSG confirmed RBD treated with of 50 mg/day 5-HTP or placebo	4-week, single-centre, randomized, double-blind placebo-controlled crossover trial	CGI and self-reported clinical status improved similarly with 5-HTP and with placebo. 5-HTP improved UPDRS part 2 score. 5-HTP produced an increase in the total percentage of stage REM sleep without a related increase of RBD episodes and with a trend toward reduction in arousal index and wake after sleep onset.	I (>50%)
**Antiepileptic drugs**					
Carbamazepine	Bamford, 2003 [76]	One patient with iRBD treated with 100 mg of carbamazepine twice daily	Single case report	75% improvement of RBD symptoms maintained for at least 14 months.	III (NA)
Levetiracetam	Batalini et al., 2016 [77]	One patient with RBD associated with probable LBD treated with 1000 mg of levetiracetam twice daily	Single case report	Reduction of RBD episodes by 50% and worsening to basal level after withdrawal of levetiracetam	III (NA)
**Cannabinoids**					
Cannabidiol	Chagas et al., 2014 [78]	4 patients with PD and RBD symptoms (*n* = 2) or PSG-confirmed RBD (*n* = 2). Treatment with 75 mg/day of cannabidiol (1 with 300 mg/day)	Case series	Marked (1) or complete (3) improvement in RBD symptoms during a 6-week treatment	III (NA)
	De Almeida et al., 2021 [79]	33 patients with PD and PSG-confirmed RBD. Treatment with 75 mg/day of cannabidiol, 300 mg/day of cannabidiol, or placebo. Assessment of the frequency of nights with RBD, CGI-I, and CGI-S.	14-week, phase II/III, double-blind, randomized, placebo-controlled clinical trial	Non-significant differences between cannabidiol and placebo in the frequency of nights with RBD, CGI-S, and CGI-I scale scores. Improvement in average sleep satisfaction from the 4th to 8th week	I (>50%)
**Herbals**					
Yokukansan (Yi-Gan San)	Shinno et al., 2008 [80]	3 patients with PSG-confirmed RBD treated with 2.5–7.5 g/day of yokukansan in 2 cases as add-on therapy to clonazepam	Case series	Complete or significant improvement of RBD symptoms in 3 patients, with relapsing in 2 patients after yokukansan withdrawal and new improvement after its reintroduction	III (NA)
	Matsui et al., 2019 [81]	36 patients with PSG-confirmed iRBD treated with yokukansan alone (*n* = 17) or as add-on therapy (*n* = 19). Assessment with CGI-I, and CGI-S.	Retrospective analysis of clinical records	70.6% of patients with yokukansan in monotherapy and 21.1% of them receiving yokukansan as add-on therapy were “responders” (“very much improved” and “much improved”). Mean reductions in CGI-S and CGI-I scores were significantly higher in patients on monotherapy.	II (NA)
	Ozone et al., 2020 [82]	23 patients with RBD treated with yokukansan (*n* = 11) or clonazepam (*n* = 12) for at least 3 months. Assessment with RBDQ-JP and SF-8 scales	Retrospective analysis of clinical records	Significant and similar improvement of RBDQ-JP score after treatment with both yokukansan (3.0 + 1.0 g/day) and with clonazepam (0.9 + 0.5 g/day) Non-significant changes in SF-8 scores with yokukansan and with clonazepam.	II (NA)

CGI-I, clinical global impression-improvement; CGI-S, clinical global impression-severity; DLB, dementia with Lewy bodies; 5-HT, 5-hydroxytryptamine (serotonin); 5-HTP, 5-hydroxytryptophan; iRBD, idiopathic or isolated REM sleep behaviour disorder; MT, melatonin; PD, Parkinson’s disease; PDD, Parkinson’s disease dementia; PSG, polysomnography; RBD, REM sleep behaviour disorder; RBDQ-JP, RBD Questionnaire Japanese version; REM, rapid eye movements; SF-8, Short-Form Health Survey; UPDRS, Unified Parkinson’s Disease Rating Scale; WASO, wake after sleep onset.

## Data Availability

Not applicable.
